# Tuberatolide B Suppresses Cancer Progression by Promoting ROS-Mediated Inhibition of STAT3 Signaling

**DOI:** 10.3390/md15030055

**Published:** 2017-02-25

**Authors:** Youn Kyung Choi, Junseong Kim, Kang Min Lee, Yu-Jeong Choi, Bo-Ram Ye, Min-Sun Kim, Seong-Gyu Ko, Seung-Hong Lee, Do-Hyung Kang, Soo-Jin Heo

**Affiliations:** 1 Jeju International Marine Science Center for Research & Education, Korea Institute of Ocean Science & Technology (KIOST), Jeju 63349, Korea; choiyk@kiost.ac.kr (Y.K.C.); junseong@kiost.ac.kr (J.K.); ramiz@kiost.ac.kr (B.-R.Y.); iskim9924@kiost.ac.kr (M.-S.K.); dohkang@kiost.ac.kr (D.-H.K.); 2 Laboratory of Clinical Biology and Pharmacogenomics, Department of Preventive Medicine, College of Korean Medicine, Kyung Hee University, Seoul 02247, Korea; linkin1102@naver.com (K.M.L.); ehowlqk11@naver.com (Y.-J.C.); epiko@khu.ac.kr (S.-G.K.); 3 Division of Food Bioscience, and Korea Nokyong Research Center, Konkuk University, Chungju 380-701, Korea; leesh80@kku.ac.kr

**Keywords:** tuberatolide B, cancer, STAT3, ROS, DNA damage

## Abstract

Tuberatolide B (TTB, C_27_H_34_O_4_) is a diastereomeric meroterpenoid isolated from the Korean marine algae *Sargassum macrocarpum*. However, the anticancer effects of TTB remain unknown. In this study, we demonstrate that TTB inhibits tumor growth in breast, lung, colon, prostate, and cervical cancer cells. To examine the mechanism by which TTB suppresses cell growth, we determined the effect of TTB on apoptosis, ROS generation, DNA damage, and signal transduction. TTB induced ROS production in MDA-MB-231, A549, and HCT116 cells. Moreover, TTB enhanced DNA damage by inducing γH2AX foci formation and the phosphorylation of DNA damage-related proteins such as Chk2 and H2AX. Furthermore, TTB selectively inhibited STAT3 activation, which resulted in a reduction in cyclin D1, MMP-9, survivin, VEGF, and IL-6. In addition, TTB-induced ROS generation caused STAT3 inhibition, DNA damage, and apoptotic cell death. Therefore, TTB suppresses cancer progression by promoting ROS-mediated inhibition of STAT3 signaling, suggesting that TTB is useful for the treatment of cancer.

## 1. Introduction

Reactive oxygen species (ROS) are produced by the reactions of oxygen-related molecules, such as peroxides, superoxide, hydroxyl radicals, and singlet oxygen [1]. ROS are also generated by the metabolism of oxygen in normal cells and have critical roles in homeostasis and cell signaling [2]. However, various diseases such as cardiovascular disease, Alzheimer’s disease, Parkinson’s disease, and cancer are caused by abnormal ROS generation [3,4,5,6]. In particular, ROS production can induce the death of cancer cells by apoptosis and DNA damage [7,8,9]. Therefore, diverse chemotherapeutic agents that increase ROS generation have been studied for cancer treatment [7,10,11]. In addition, excessive ROS affects various signaling pathways in cancer cells, including Akt, ERK, JNK, p38, NFκB, and STAT3 [12,13,14]. Signal transducer and activator of transcription 3 (STAT3) is a member of the STAT family, which is important for development, apoptosis, proliferation, cell cycle progression, angiogenesis, inflammation, and cancer metastasis [15,16,17,18]. Moreover, STAT3 is frequently activated in nearly 70% of different cancer types, including breast, lung, colon, prostate, cervical, renal, pancreatic, and ovarian cancers [19,20,21,22,23]. Accordingly, STAT3 is a potential therapeutic target for cancer treatment, and many STAT3 inhibitors, including synthetic drugs, anti-sense oligonucleotides targeting STAT3, and small molecules derived from natural sources, have been developed to inhibit deregulated STAT3 signaling cascades in cancer [24,25]. 

Tuberatolide B (TTB, C27H34O4) is a diastereomeric meroterpenoid isolated from the Korean marine algae *Sargassum macrocarpum* and acts as a Farnesoid X receptor (FXR) antagonist [26]. However, the effect of TTB on various diseases, including cancer, remains unknown. Here, we report that TTB inhibits cancer growth by promoting ROS-mediated inhibition of STAT3 signaling and inducing DNA damage, thereby suggesting that TTB is useful for the treatment of cancer. 

## 2. Results

### 2.1. TTB Induces Apoptosis in Cancer Cells 

**Figure 1:**
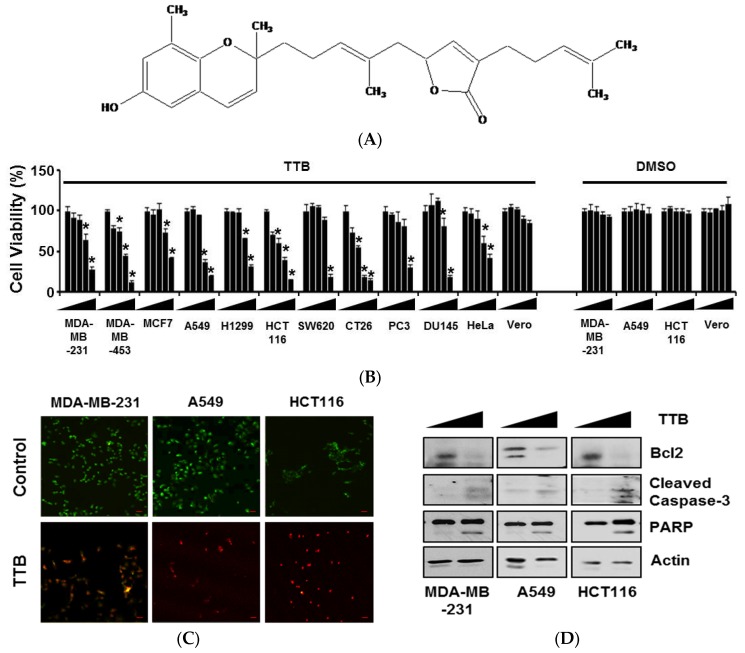

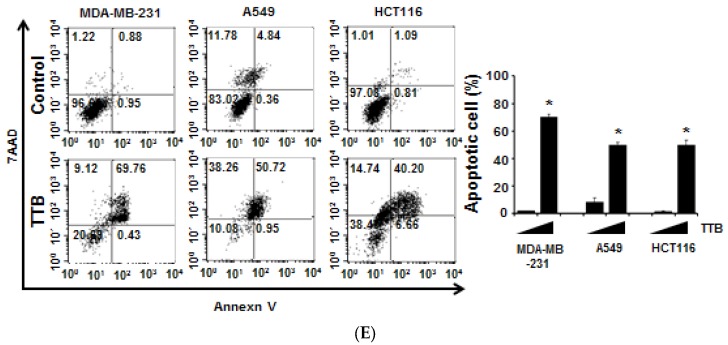
Tuberatolide B (TTB) induces apoptotic cell death: (**A**) The chemical structure of TTB; (**B**) Cells were seeded in 96-well plates and then treated with various concentrations of TTB (0, 10, 25, 50, and 100 μM) and DMSO for 48 h. Experiments were performed three times. * *p* < 0.05; (**C**) Cells were treated with 100 μM of TTB for 8 h, and cell death was determined using a live and dead cell assay kit. Red fluorescence-positive cells were considered dead cells. The object was 20× and scale bar indicates 10 μm; (**D**) Cells were treated with 100 μM of TTB for 8 h and then subjected to western blotting for apoptosis-related molecules, including Bcl2, cleaved caspase-3, and PARP. Actin was used as an internal control; (**E**) MDA-MB-231, A549, and HCT116 cells were treated with TTB (100 μM) for 48 h and then harvested. Cells were stained with annexin V and 7AAD in binding buffer at room temperature in the dark. Stained cells were detected by FACSCalibur. The graph shows examples of annexin V only-positive cells (early apoptotic cells) and annexin V and 7AAD double-positive cells (late apoptotic cells) from the total stained cells. * *p* < 0.05. Data are shown as the mean of three independent experiments, and the error bars represent the mean ± standard deviation (SD).

Various cancer cell lines, including breast cancer (MDA-MB-231, MDA-MB-453, and MCF7), lung cancer (A549 and H1299), colon cancer (HCT116, SW620, and CT26), prostate cancer (PC3 and DU145), cervical cancer (HeLa) and non-malignant normal Vero cells were treated with different concentrations (0, 10, 25, 50, and 100 μM) of TTB for 48 h. TTB suppressed cancer cell viability. In addition, TTB did not affect normal monkey kidney epithelial cell viabilities (Figure 1B). In the live and dead assay, TTB increased the number of dead cells (Figure 1C). To examine if TTB inhibited cell growth by inducing apoptotic cell death, we investigated the expression of apoptosis-related proteins and the extent of annexin V staining using western blot and flow cytometry, respectively. TTB decreased the expression of Bcl2 and increased the cleavage of caspase-3 and PARP (Figure 1D). In additions, TTB enhanced the percentage of annexin V-positive apoptotic cells (Figure 1E). Thus, TTB inhibits cancer cell growth by inducing apoptotic cell death.

### 2.2. TTB Increases ROS Generation in Cancer Cells 

**Figure 2:**
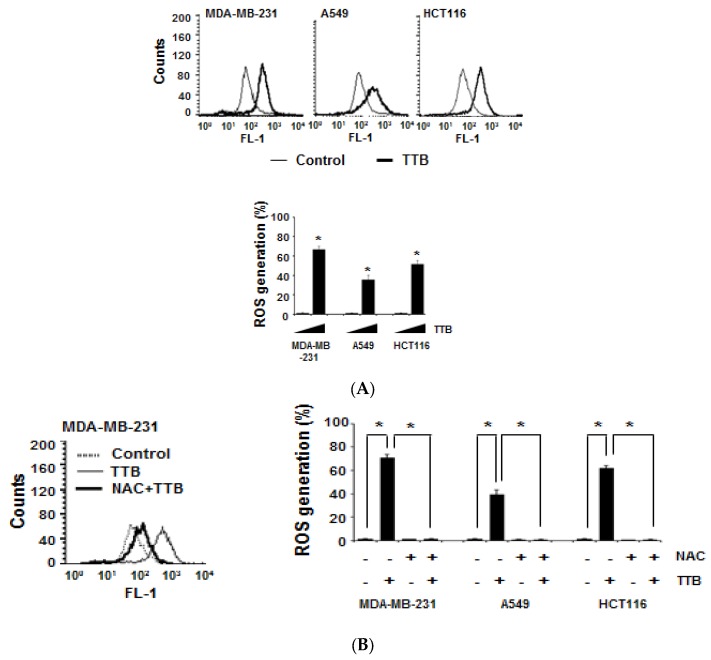
TTB increases reactive oxygen species (ROS) generation in cancer cells: (**A**) MDA-MB-231, A549 and HCT116 cells were co-treated with 100 μM of TTB and H2DCFDA dye for 1 h at 37 °C. ROS production was detected by FACSCalibur. Graph shows H2DCFDA-positive cells from the total cells. * *p* < 0.05; (**B**) Cells were pretreated for 1 h with or without *N*-acetyl-l-cysteine (NAC), followed by exposure to 100 μM of TTB and H2DCFDA dye for 1 h at 37 °C. * *p* < 0.05. Data are shown as the mean of three independent experiments (error bars are mean ± standard deviation (SD)).

The regulation of intracellular ROS generation plays a role in many cellular functions, such as cell proliferation and apoptosis, which are critical processes in tumor development. To investigate the effect of TTB on ROS production, we assessed ROS generation using flow cytometry. When compared to the control, TTB increased ROS generation by approximately 67%, 36%, and 52% in MDA-MB-231, A549, and HCT116 cells, respectively (Figure 2A). Moreover, the well-known ROS scavenger NAC suppressed TTB-mediated ROS production in cancer cells (Figure 2B). Therefore, TTB induces ROS generation in many types of cancer cells.

### 2.3. TTB Induces DNA Damage in Cancer Cells

**Figure 3:**
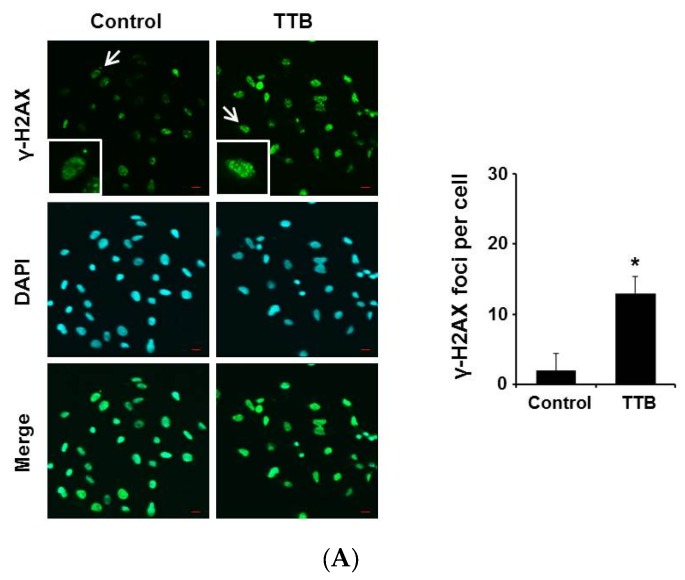

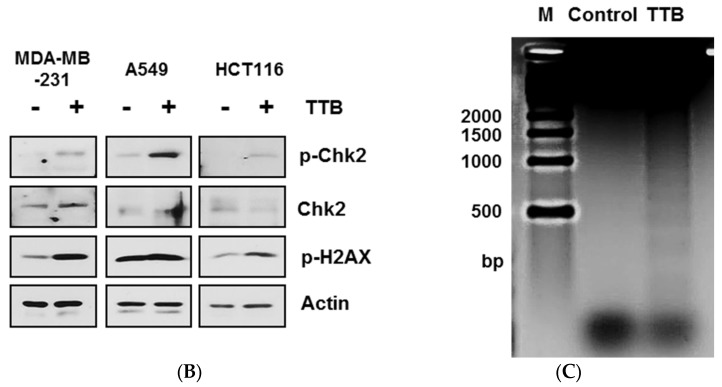
TTB induces DNA damage in cancer cells: (**A**) MDA-MB-231 cells were treated with 100 μM of TTB for 24 h and stained with anti-γH2AX (1:200) primary antibody and Alexa-Fluor-488 (1:200) secondary antibody. For the counter staining, DAPI was used to stain the nucleus. Images were acquired with Olympus FV10i Self Contained Confocal Laser System. The object was 20× and scale bar indicates 10μm. Graph shows γH2AX-positive cells from the total cells; (**B**) MDA-MB-231, A549 and HCT116 cells were treated with TTB (100 μM) for 8 h and then performed western blots with anti-p-Chk2, -Chk2 and γ-H2AX. Actin used for loading control; (**C**) MDA-MB-231 cells were treated with 100 μM of TTB for 24 h and then DNA fragmentation assay was performed. Experiments were performed in triplicate. Bar indicate means and standard deviations.

DNA damage-inducing drugs that cause the apoptotic cell death of cancer cells may be a viable cancer treatment [27,28]. Therefore, we examined effect of TTB on DNA damage. γ-H2AX staining is a well-known marker of oxidative-related DNA damage [29,30]. TTB increased co-staining of DAPI and γH2AX foci in MDA-MB-231 cells (Figure 3A). In additions, TTB induced the phosphorylation of Chk2 and H2AX in MDA-MB-231, A549 and HCT-116 cells (Figure 3B). Moreover TTB enhanced DNA fragmentation in MDA-MB-231 cells when compared with the control (Figure 3C). Thus, TTB increases DNA damage in cancer cells.

### 2.4. TTB Selectively Inhibits the STAT3 Signaling Pathway in Cancer Cells 

**Figure 4:**
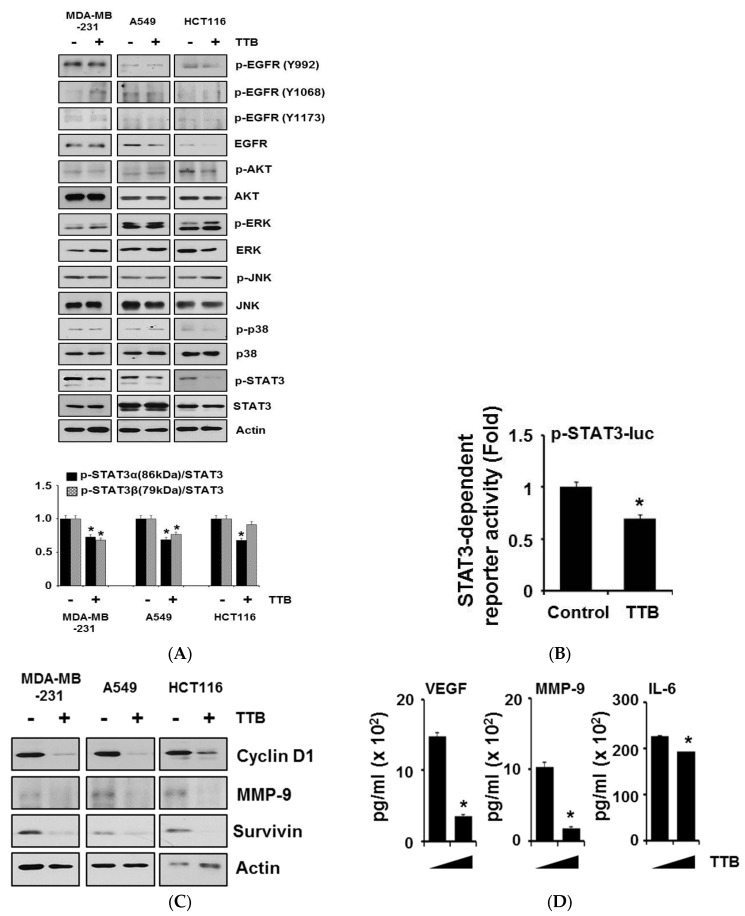
TTB selectively suppresses STAT3 signaling pathway: (**A**) MDA-MB-231, A549 and HCT116 cells were treated with TTB (100 μM) for 15 min and whole lysates were analyzed by western blot with anti-p-EGFR (Y992), -p-EGFR (Y1068), -p-EGFR (Y1173), -EGFR, -p-AKT, -AKT, -p-ERK, -ERK, -p-JNK, -JNK, -p-p38, -p38, -p-STAT3 and -STAT3. Actin was used for internal control. Quantitative analyses of p-STAT3 expression was performed using the Image J software; (**B**) MDA-MB-231 cells were transfected with the STAT3-dependent luciferase reporter and then treated with TTB (100 μM) for 6 h. Luciferase assay were done by using dual-luciferase reporter assays. All transfections included the RLTK-Luc for transfection efficiency. * *p* < 0.05; (**C**) Cells were treated with 100 μM of TTB for 24 h and performed western blots with STAT3 target genes, such as Cyclin D1, MMP-9 and Survivin. Actin was used as loading control; (**D**) MDA-MB-231 cells were treated with TTB (100 μM) for 24 h and supernatants were collected. Secreted expression levels of VEGF, MMP-9 and IL-6 were detected by ELISA assay. * *p* < 0.05. Experiments were performed in triplicate. Bars indicate means and standard deviations.

To further elucidate the anticancer effects of TTB on cancer cells, we identified which intracellular signaling pathways were involved. Cells were treated with 100 μM TTB for 15 min and then subjected to western blotting. STAT3 phosphorylation was selectively and strongly suppressed by TTB. The phosphorylation of EGFR (Y992, Y1068, and Y1173), AKT, ERK, JNK, and p38 remained unchanged after TTB treatment (Figure 4A). Next, to confirm the TTB-mediated inhibition of the STAT3 pathway, we examined the transcriptional activation of STAT3 using a luciferase assay. As shown in Figure 4B, TTB reduced STAT3 transcriptional activity when compared with the control. Next, we assessed the effect of TTB on the expression of STAT3-target genes. TTB decreased the protein expression levels of many STAT3-target genes, including cyclin D1, MMP-9 and survivin (Figure 4C). TTB also reduced the secreted protein levels of VEGF, MMP-9, and IL-6 in MDA-MB-231 cells (Figure 4D). Thus, our data suggest that TTB selectively suppresses STAT3 activity and STAT3-dependent gene expression. 

### 2.5. TTB-Induced ROS Generation Causes STAT3 Inhibition and Apoptosis

**Figure 5:**
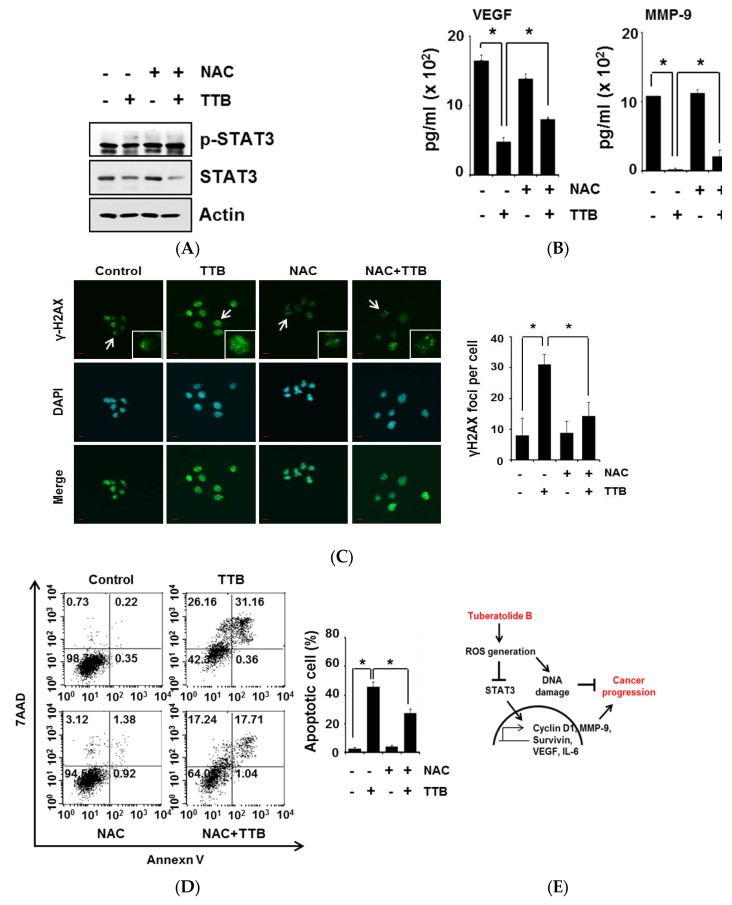
TTB-induced ROS generation causes STAT3 inhibition and apoptosis: (**A**) MDA-MB-231 cells were pretreated with NAC (2.5 mM) for 1 h and then exposed to TTB (100 μM) for 15 min. p-STAT3 and STAT3 protein expression levels were analyzed by western blot. Actin used for loading control; (**B**) MDA-MB-231 cells were pretreated for 1 h with or without NAC, followed by exposure to 100 μM of TTB for 24 h and then harvested culture media. VEGF, MMP-9 and IL-6 levels were analyzed with ELISA assay. Experiments were performed in triplicate. Bar indicate means and standard deviations; (**C**) MDA-MB-231 cells were pretreated with NAC for 1 h and then treated with 100 μM of TTB for 8 h. Cells were stained with anti-γH2AX antibody and DAPI was used for nuclear staining. Images were obtained with using Olympys FV10i Self Contained Confocal Laser System. The object was 20× and scale bar indicates 10 μm. * *p* < 0.05; (**D**) HCT116 cells were pretreated with NAC for 1 h and then exposed to TTB (100 μM) for 48 h. Cells were stained with Annexin V and 7AAD at room temperature in the dark. Experiments were performed in triplicate. Bar indicate means and standard deviations. * *p* < 0.05; (**E**) A schematic representation of the mechanisms for TTB suppressed cancer cell growth.

ROS generation inhibits STAT3 signaling [14,31]; thus, we examined STAT3 expression in cells after co-treatment with TTB and NAC. As shown in Figure 5A, the TTB-mediated reduction in phosphorylated STAT3 was restored after treatment with NAC. Furthermore, co-treatment with TTB and NAC increased STAT3-dependent gene expression (VEGF and MMP-9) when compared with the TTB treatment, suggesting that the inhibition of STAT3 pathway activation by TTB was at least partially attributed to ROS generation in MDA-MB-231 cells (Figure 5B). ROS generation induces DNA damage [32]; thus, we evaluated the role of ROS in TTB-induced DNA damage by treating cells with NAC. Indeed, TTB-induced γH2AX foci were abolished in the presence this ROS inhibitor (Figure 5C). In addition, NAC reduced TTB-mediated apoptotic cell death (Figure 5D). Therefore, TTB-induced DNA damage, apoptosis and reduced STAT3 activity occurs via ROS generation in cancer cells.

## 3. Discussion

Cancer still remains a deadly disease and has a high incidence and death rate worldwide [25]. Unlike normal cells, cancer cells have some characteristics, such as sustained proliferative signaling, evaded growth suppressors, activated invasion, and metastasis, that enable replicative immortality, induce angiogenesis, and resist cell death [33]. Therefore, targeted cancer therapeutic agents are developed for cancer patients for a long time [34]. Especially because constitutive STAT3 activation is associated with poor prognosis in cancer patients, STAT3 has been investigated as a cancer therapeutic target [35,36]. Therefore, STAT3 specific inhibitor may be useful cancer treatment and many STAT3 inhibitors are in the process of being tested in clinical trials [36]. Phosphorylated tyrosine-705 STAT3 (Y-705) is required for STAT3 dimerization and nuclear translocation. Dimeric STAT3 bind to specific DNA response elements in the promoters of target genes [37]. In our study, TTB selectively suppressed STAT3 phosphorylation, transcriptional activity and expression of target genes such as Cyclin D1, MMP-9, Survivin, and IL-6. 

Our data indicate that TTB induction of ROS level is a key modulate to enhance the apoptosis in various cancer cells. In addition, NAC rescued TTB-mediated apoptosis. ROS can be generated from exogenous sources, such as chemical, pharmaceutical, and endogenous sources, including mitochondria, activation of inflammatory cells, and peroxisomes [38,39]. Importantly, numerous studies have shown that many cancer chemotherapeutic drugs have anticancer effects by inducing ROS-mediated apoptosis. For example, the classic anticancer drugs adriamycin and cisplatin induces excessive levels of ROS, resulting in DNA damage and apoptotic cell death [39]. Moreover, a number of natural compounds, such as tocopheryl succinate (a vitamin E analog), c-phycocyanin (a major phycobiliprotein from blue-green algae), and β-phenylethyl isothiocyanate (PEITC), are reported to induce ROS production and kill cancer cells [7,40,41]. Thus, ROS is crucial for inducing cell death in cancer cells. Moreover, ROS are inducing DNA damage, resulting in single- or double-strand breakage, DNA cross-linking, and base modification, and these events can result in cell death [42]. DNA double-strand breaks (DSBs) induce H2AX phosphorylation on serime 139, then called gamma-H2AX (γ-H2AX) and γ-H2AX foci formation at DSB sites occurs rapidly [43,44]. Furthermore serin/threonine kinase Chk2 is a major regulator of the DNA damage response [45] and phosphorylated Chk2 is essential for H2AX phosphorylation [46]. In our study, TTB induced γ-H2AX foci formation and increased phosphorylation of Chk2 and H2AX levels. Moreover, TTB enhanced DNA fragmentation. In additions, the ROS scavenger NAC inhibited TTB-mediated γ-H2AX foci formation and apoptotic cell death in cancer cells.

## 4. Materials and Methods

### 4.1. Extraction and Isolation of TTB

The brown alga *Sargassum macrocarpum* was collected from along the coast of Jeju Island, Korea. The sample was washed thrice with tap water to remove salt, sand, and epiphytes attached to its surface, followed by careful rinsing with fresh water and freezing in a medical refrigerator at −20 °C. Thereafter, the frozen sample was lyophilized and homogenized with a grinder prior to extraction. All chemicals and reagents used were of analytical quality and sourced from trusted commercial sources (grade ≥ 95%). The dried *S. macrocarpum* powder was extracted thrice with 80% aqueous methanol at the room temperature. The liquid layer was obtained via filtration, and the filtrate was concentrated by using an evaporator under reduced pressure. The extract was suspended in water, and the aqueous layer was partitioned with chloroform. Then, the chloroform fraction was fractionated by silica column chromatography with stepwise elution of chloroform–methanol mixture (50:1→1:1) to separate active fractions in chloroform extract. A combined active fraction was further subjected to a Sephadex LH-20 column saturated with 100% methanol, and then purified by reversed phase high performance liquid chromatography (HPLC) using a Waters HPLC system (Alliance 2690; Waters Corp., Milford, MA, USA) equipped with a Waters 996 photodiode array detector and C18 column (J’sphere ODS-H80, 250 × 4.6 mm, 4 μm; YMC Co., Kyoto, Japan) by stepwise elution with methanol–water gradient (UV range, 220 nm; flow rate, 1 mL/min). Finally, the purified compound was identified by comparing its 1H and 13C NMR data with literature [26]. The chemical structure of tuberatolide B is indicated in Figure 1. The compound was dissolved in dimethylsulfoxide (DMSO) and employed in experiments in which the final concentration of DMSO in culture medium was adjusted to <0.01%.

### 4.2. Cell Lines and Cell Cultures

Breast cancer cells (MDA-MB-231, MDA-MB-453, and MCF7), Lung cancer cells (A549 and H1299), Colon cancer cells (HCT116, SW620, and CT26), Prostate cancer cells (PC3 and DU145), and Cervical cancer cells (HeLa) were purchased from Korean Cell Line Bank (KCLB, Seoul, Korea). MDA-MB-231, MCF-7 and HeLa cells were cultured in DMEM medium with 10% fetal bovine serum (FBS) and 1% antibiotics. MDA-MB-453, A549, H1299, HCT116, SW620, CT26, PC3, and DU145 cells were maintained in RPMI-medium supplemented with 10% fetal bovine serum and 1% antibiotics.

### 4.3. Cell Viability and Apoptotic Analysis

Various cancer cells were seeded on 96-well plates and treated with TTB for 48 h. Cell viability was determined using the MTT assay (Sigma-Aldrich, St. Louis, MO, USA). Absorbance was read at 570 nm on the ELISA reader (Molecular Devices, Palo Alto, CA, USA). Cells were treated with TTB for 48 h and then resuspended in binding buffer. After cells were stained with Annexin V-FITC (BD Bioscience, San Jose, CA, USA) in the dark at room temperature for 15 min, Annexin V-stained cells were incubated with 7AAD in the dark at room temperature for 15 min. Annexin V- and 7AAD-positive cells were detected by FACSCalibur flow cytometry (BD Bioscience, San Jose, CA, USA). Live and dead assay was performed with the live and dead cell assay kit (Abcam, Cambridge, UK) according to the manufacturer’s instruction. 

### 4.4. Western Blot and Immunocytochemistry

Cells were lyzed with RIPA buffer and equal amount of protein in total cell lysate was run on 8% to 12% sodium dodecyl sulfate polyacrylamide gel electrophoresis (SDS-PAGE) and transferred to nitrocellulose membranes. Membrane was blocked and blotted with the relevant primary antibodies. Anti-Actin, -Bcl2, -Chk2, -ERK2, -p-ERK, -p-JNK and -p-p38 antibodies were purchased from Santa Cruz Biotechnology (Santa, CA, USA). Anti-AKT, -cleaved caspase-3, -EGFR, -γH2AX, -JNK1, -p38, -p-AKT, -PARP, -p-chk2, p-EGFR, p-STAT3, and -STAT3 antibodies were obtained from Cell Signaling (Danvers, MA, USA). For γH2AX formation, cells were seeded in 6-well plates with coverglasses and treated with TTB for 24 h. Cells were fixed with 4% paraformaldehyde for 15 min and permeabilized with 0.5% Triton X-100 for 7 min. After being blocked with 10% FBS and 1% BSA in 0.1% Tween-20 buffer, cells were stained with 1:200 of γH2AX primary antibody and 1:100 of Alexa-Fluor-488 secondary antibody (Invitrogen, San Diego, CA, USA). For the counter staining, DAPI was used for nucleus staining. Images were acquired with Olympus FV10i Self Contained Confocal Laser System.

### 4.5. ROS Measurement and DNA Fragmentation Assay 

Cells were seeded in 6-well plates and treated with TTB and H2DCFDA for 1 h at 37 °C. After harvested, the data was analyzed by FACSCalibur flow cytometry measuring by the FL1 channel. For the inhibition of ROS generation, cells were pretreated with *N*-acetyl-l-cysteine (NAC, 2.5 mM, Sigma-Aldrich, St. Louis, MO, USA) for 1 h before TTB and H2DCFDA co-treatment. The data was analyzed by FACSCalibur flow cytometry. For DNA fragmentation, cells were seed in 100 mm dishes and treated with TTB for 24 h. After harvest, cells were lyzed with DNA isolation buffer (0.1 M NaCl, 0.01M EDTA, 0.3M Tris-HCl (pH 7.5) and 0.2 M sucrose) and DNA gel electrophoresis was performed. 

### 4.6. Luciferase Assay and ELISA

Cells were seeded in 24-well plates and p4xM67-TK-luc plasmid (Addgene, Cambridge, MA, USA) was transfected in MDA-MB-231 cells by using Lipofectamine reagent (Invitrogen, Carlsbad, CA, USA). Cells were treated with TTB for 6 h, and then luciferase assay was performed by using dual-luciferase reporter assay kits (Promega, Madison, WI, USA) according to the manufacturer’s instructions. All transfections included the RLTK-Luc (kindly provide by Sang Hoon Kim) for transfection efficiency. For ELISA assay, cells were seeded in 6-well plates and treated with TTB. After 24 h, supernatants were harvested and secreted protein levels of VEGF, MMP-9, and IL-6 were performed with human VEGF and MMP-9 ELISA kits (R&D Systems, Minneapolis, MN, USA) and human IL-6 ELISA kit (BD Biosciences, San Jose, CA, USA) according to the manufacturer’s instructions.

### 4.7. Statistics

All the data were performed in triplicate, and shown as means and standard deviations. *p*-values less than 0.05 in the two-tailed Student’s *t*-test were considered significant.

## 5. Conclusions

In conclusion, we provide evidence for the first time that anticancer effect of TTB on diverse cancer cells result from the induction of ROS-mediated apoptosis by inhibiting of STAT3 phosphorylation and enhancing of DNA damage. Therefore, TTB might be an effective and useful chemotherapy agent against cancer. 

## Supplementary Materials

The following are available online at www.mdpi.com/1660-3397/15/3/55/s1. Figure S1: EC_50_ values of TTB in cancer cell lines (MDA-MB-231, MDA-MB-453, MCF7, A549, H1299, HCT116, SW620, CT26, PC3, DU145 and HeLa), Table S1: EC50 values of Tuberatolide B (TTB) in cancer cell lines (MDA-MB-231, MDA-MB-453, MCF7, A549, H1299, HCT116, SW620, CT26, PC3, DU145 and HeLa).
