# Supplementary material for: Tuberatolide B Suppresses Cancer Progression by Promoting ROS-Mediated Inhibition of STAT3 Signaling
Source: Mar Drugs. 2017 Feb 25;15(3):55. doi: 10.3390/md15030055 (PMC5367012; doi:10.3390/md15030055)
Supplement: Supplementary file 1 [file marinedrugs-15-00055-s001.pdf]

Supplementary Materials: Tuberatolide B Suppresses Cancer Progression by Promoting ROS-Mediated Inhibition of STAT3 Signaling

Youn Kyung Choi, Junseong Kim, Kang Min Lee, Yu-Jeong Choi, Bo-Ram Ye, Min-Sun Kim, Seong-Gyu Ko, Seung-Hong Lee, Do-Hyung Kang and Soo-Jin Heo

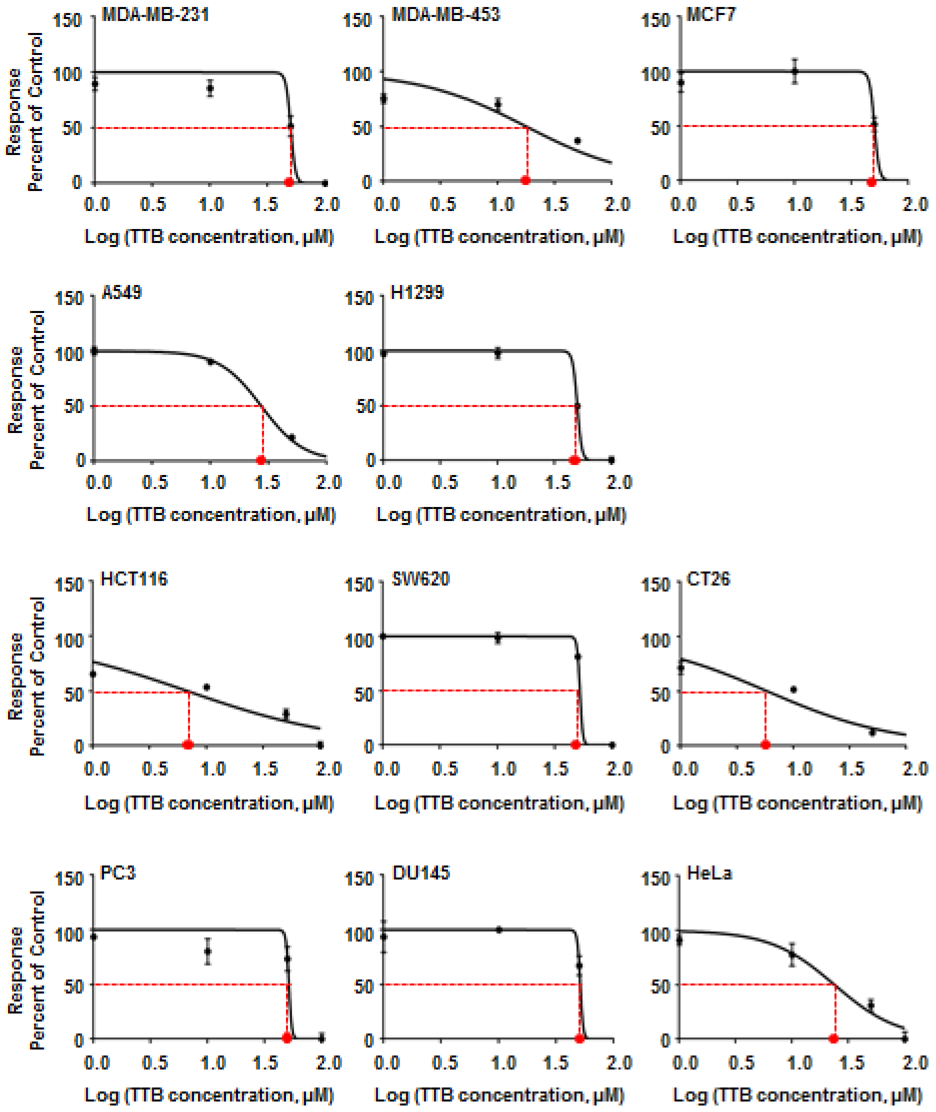

Figure S1. EC_{50} curves of Tuberatolide B (TTB) in cancer cell lines (MDA-MB-231, MDA-MB-453, MCF7, A549, H1299, HCT116, SW620, CT26, PC3, DU145 and HeLa). EC_{50} curves were analyzed using GraphPad Prism v7 (GraphPad Prism Software, CA, USA, 2016).

Table S1. EC_{50} values of Tuberatolide B (TTB) in cancer cell lines (MDA-MB-231, MDA-MB-453, MCF7, A549, H1299, HCT116, SW620, CT26, PC3, DU145 and HeLa). EC_{50} values were analyzed using GraphPad Prism v7 (GraphPad Prism Software, CA, USA, 2016).

Organ	Cell line	EC50 (log, μM)	EC50 (μM)
**Breast cancer**	MDA-MB-231	~1.7	~50.14
	MDA-MB-453	1.25	17.77
	MCF7	~1.7	~50.14
**Lung cancer**	A549	1.438	27.44
	H1299	~1.699	~49.97
**Colon cancer**	HCT116	0.8319	6.791
	SW620	~1.717	~52.15
	CT26	0.7688	5.873
**Prostate cancer**	PC3	~1.712	~51.51
	DU145	~1.708	~51.06
**Cervical cancer**	HeLa	1.376	23.78
